# Sex differences and the role of education in cognitive ageing: analysis of two UK-based prospective cohort studies

**DOI:** 10.1016/S2468-2667(20)30258-9

**Published:** 2021-01-28

**Authors:** Mikaela Bloomberg, Aline Dugravot, Julien Dumurgier, Mika Kivimaki, Aurore Fayosse, Andrew Steptoe, Annie Britton, Archana Singh-Manoux, Séverine Sabia

**Affiliations:** a Department of Epidemiology and Public Health, University College London, London, UK; b Epidemiology of Ageing and Neurodegenerative Diseases, Université de Paris, Inserm U1153, Paris, France; c Cognitive Neurology Centre, Saint-Louis Lariboisière Fernand-Widal Hospital, AP-HP, Université de Paris, Paris, France; d Department of Behavioural Science and Health, University College London, London, UK

## Abstract

**Background:**

Previous studies have shown an excess risk of Alzheimer's disease and related dementias among women. Education is thought to have a causal association with dementia onset. We aimed to investigate the role of education in influencing sex differences in cognitive ageing.

**Methods:**

We analysed data from two prospective cohort studies in the UK; the English Longitudinal Study of Ageing (ELSA) and the Whitehall II study, to assess sex differences in cognitive performance and cognitive decline by birth cohort (birth year 1930–38, 1939–45, or 1946–55), before and after adjustment for education, and by high and low education level. Memory was assessed using immediate recall, for which data were available from all waves of the ELSA (2002–14) and Whitehall II (1997–2015) studies. Fluency was assessed using a semantic fluency test based on an animal naming task, with data available from all waves of the Whitehall II study and waves one to five (2002–10) and wave seven (2014) of the ELSA study. Cognitive scores were standardised separately in each study based on the mean and SD of the corresponding test among participants aged 50–59 years with secondary education.

**Findings:**

15 924 participants were included from the two studies. In pooled analyses, women had better memory scores than men in all birth cohorts, irrespective of adjustment for education (eg, at age 60 years, birth cohort 1930–38, mean difference between sexes [male scores minus female scores] –0·25 SDs [95% CI –0·32 to –0·19] after adjustment for education), and in both education level groups. Memory decline was faster in men than in women (at age 60 years, birth cohort 1946–55, mean difference in 13-year change –0·15 SDs [–0·20 to –0·09]; after adjustment for education –0·14 SDs [–0·20 to –0·08]). Men had better fluency scores than women in earlier birth cohorts and in the low education group (at age 60 years, birth cohort 1930–38, mean difference 0·20 SDs [95% CI 0·05 to 0·36]); but women had better fluency scores than men in later birth cohorts and in the high education group (at age 60 years, birth cohort 1946–55, mean difference –0·17 SDs [–0·24 to –0·10]). No sex differences were observed for fluency decline.

**Interpretation:**

Our findings suggest that decreasing disparities between sexes in education, due to secular increases in educational opportunities, could attenuate sex differences in dementia risk and cognitive decline in the future.

**Funding:**

National Institute on Aging, National Institutes of Health; UK Medical Research Council; British Heart Foundation; and National Institute for Health Research.

## Introduction

Alzheimer's disease and related dementias are leading causes of disability in adults aged 70 years or older^1^ and appear to disproportionately affect women,^2^, ^3^ with a 50% greater risk in women than in men reported in a meta-analysis of 12 studies.^4^ Although sex differences in the risk of Alzheimer's disease and related dementias might have a biological basis,^2^, ^3^ it is possible that socially constructed gender norms—for example, in relation to access to education—also affect this risk.^5^ Societal expectations of gender roles have historically manifested in reduced access to higher education for women.^5^ Observational studies have suggested a causal role of education in delaying the onset of Alzheimer's disease due to its effect on cognitive reserve,^2^, ^3^, ^6^ which refers to the ability of the brain to mitigate the clinical manifestation of neuropathological damages.^7^ Higher education level is associated with better cognitive performance in several cognitive domains,^8^ including memory, fluency, and executive function. As the association between cognitive performance and risk of Alzheimer's disease and related dementias is well established,^9^ disparities between sexes in education are thus a potential pathway through which differences between sexes in the risk of Alzheimer's disease and related dementias are generated.^2^, ^3^, ^10^

Most studies have adjusted for the effect of education in analyses of sex differences in cognitive outcomes. The few longitudinal studies that explicitly examined the effect of education on sex differences in cognitive ageing have had small sample sizes.^11^, ^12^, ^13^ Studies of how secular changes in access to education across birth cohorts affect sex differences in cognitive ageing trajectories are scarce.^14^, ^15^ Furthermore, it is probable that sex differences in cognitive ageing patterns differ between cognitive domains. Previous studies have suggested female performance advantages in memory,^16^ but have shown mixed results for other cognitive domains.^16^, ^17^, ^18^

**Research in context**

**Evidence before this study**

We searched PubMed on June 30, 2020, for publications in English from database inception until June 30, 2020, using the Medical Subject Headings search terms “sex factors”, “education”, “cognition”, “Alzheimer's disease”, “dementia”, “cognitive aging”, “aging”, “birth cohort”, and “neuropsychological test”. Previous studies have shown a greater risk of Alzheimer's disease in women than in men, and education is thought to have a causal association with cognitive performance and in delaying the onset of dementia. Although there have been some studies that investigated the role of education in sex differences in cognitive performance and decline, these studies were not sufficiently large to comprehensively assess sex differences in cognitive outcomes and did not examine the role of secular changes in education in men and women.

**Added value of this study**

The identification of factors that affect adult cognitive performance and rate of cognitive decline is important to develop interventions that offer protection against adverse cognitive outcomes, such as Alzheimer's disease and related dementias, at older ages. Sex differences in cognitive outcomes in older adults (aged 65 years or older) have often been examined simply as the excess risk of Alzheimer's disease and related dementias in women. Our analysis of level of education and birth cohort shows that sex differences in cognitive performance are dynamic, whereby women in later birth cohorts increasingly have better memory scores, and the deficit in fluency has progressively been attenuated such that women in the high education group from later birth cohorts have higher fluency scores than men in the same birth cohort and education group. These findings need to be considered bearing in mind that studies showing excess risk of Alzheimer's disease and related dementias in women primarily involved people born in the 1920s or earlier.

**Implications of all the available evidence**

The reduction of disparities in level of education between sexes due to secular increases in access to education could change the observed excess risk of Alzheimer's disease and related dementias in women in the future. Our findings support public policies that aim to reduce sex disparities in access to education to improve cognitive outcomes in women at older ages.

Considering the complex interplay between sex, level of education, and birth cohort in shaping cognitive ageing trajectories, we used longitudinal data on men and women born between 1930 and 1955 in analyses stratified by birth cohort to examine the role of education in sex differences in memory and fluency performance and decline. The primary hypothesis was that male advantages in cognition would be attenuated in later birth cohorts due to secular changes in access to education.

## Methods

### Data sources and participants

We analysed data from two prospective cohort studies in the UK; the English Longitudinal Study of Ageing (ELSA) and the Whitehall II study. ELSA included adults in England aged 50 years and older, recruited from the 1998–2001 Health Survey for England, who have sociodemographic characteristics that are broadly representative of the general population of England.^19^ Data collection was done in 2002 (March, 2002, to March, 2003) with follow-up in 2004 (June, 2004, to July, 2005), 2006 (May, 2006, to August, 2007), 2008 (June, 2008, to July, 2009), 2010 (July, 2010, to June, 2011), 2012 (May, 2012, to May, 2013), and 2014 (June, 2014, to May, 2015). We used these seven waves of data (2002–14), which comprise participants born in 1930 and thereafter, in our analyses to harmonise the range in birth year cohorts in both studies.

Whitehall II is an ongoing cohort study of civil servants in the UK aged 35–55 years at recruitment, recruited from London-based offices from Aug 27, 1985, to March 17, 1988.^20^ Participants underwent a clinical examination every 4–5 years. A battery of cognitive tests was introduced to the study in 1997 (April 24, 1997, to Jan 8, 1999; the baseline for our analysis) and was repeated in 2002 (Oct 8, 2002, to Sept 10, 2004), 2007 (Oct 10, 2007, to Nov 18, 2009), 2012 (Jan 27, 2012, to Oct 30, 2013), and 2015 (Feb 2, 2015, to Dec 9, 2016). The Whitehall II study was approved most recently by the National Health Service (NHS) London–Harrow Research Ethics Committee (reference 85/0938). Written informed consent for participation was obtained at each contact. The ELSA study was done in accordance with the Declaration of Helsinki and ethical approval and experimental protocols were granted by NHS Research Ethics Committees under the National Research and Ethics Service. Participants provided informed written consent to the investigation.

### Procedures

The ELSA and Whitehall II studies include participants with similar sociodemographic data, as both studies were based in the UK, during the same time period.^19^, ^20^

Sex was measured in the ELSA cohort on the basis of administrative data from the Health Survey for England, and in Whitehall II from the British civil service.

Level of education was measured by questionnaire, comprising four categories (details of harmonisation are shown in the appendix [p 2]): no school qualifications, some high school qualifications (O-levels), high school diploma (A-levels), or university degree or higher. For analyses stratified by education, we used a two-category variable: low (less than A-level qualifications) or high (A-level or higher) education level. For participants with missing data on education, level of education was imputed using single imputation based on sex, birth cohort, and social class. The choice of single rather than multiple imputation was primarily to enable analyses stratified by education as each imputed dataset would yield different numbers in each education category, changing the results of stratified analyses between imputations. However, single imputation can potentially underestimate SEs and therefore result in overly precise CIs.

Birth cohorts were defined on the basis of sociohistorical events,^15^ as follows: the Depression-era cohort (birth year 1930–38), the World War 2 cohort (1939–45), and the post-War cohort (1946–55).

Other covariates included age, ethnicity (white and non-white), and practice effect (to account for changes in test performance attributed to increasing familiarity with test instruments and protocols)^15^ using a dichotomous indicator of whether or not the measure of cognitive function was the first cognitive assessment for the participant.

### Outcomes

The primary outcomes were performance and decline in memory and fluency. Memory was assessed using immediate recall. In the ELSA study, participants were read a ten-word list at 2-s intervals by an interviewer. The respondent was then asked to recall aloud as many words as possible within 2 min. In the Whitehall II study, participants listened to a list of 20 words at 2-s intervals on a tape and were then asked to recall in writing as many as possible within 2 min. Data from memory assessments were available at all waves of the ELSA (2002–14) and Whitehall II (1997–2015) studies.

Fluency was assessed using a semantic fluency test based on an animal naming task. In ELSA, participants had 1 min to say as many animals as possible aloud to an interviewer. In Whitehall II, participants were given 1 min to write as many animals as possible. Fluency was available at all waves of the Whitehall II study and waves one to five (2002–10) and wave seven (2014) of the ELSA study.

### Statistical analysis

Participant characteristics by sex and birth cohort were described separately for ELSA and Whitehall II, as well as in the pooled database. Differences between sexes in categorical variables were assessed using Pearson's χ^2^ test and in continuous variables using the *t* test. The χ^2^ trend test was used to assess birth cohort differences in participant characteristics separately in men and women.

To harmonise cognitive tests between the two studies and allow comparison between cognitive domains, cognitive scores were standardised separately in each study on the basis of the mean and SD of the corresponding test among participants aged 50–59 years with secondary education. These data were then pooled for analyses. Linear mixed models were used to assess differences between sexes in cognitive performance and decline. These models use all available data over the follow-up, handle differences in length of follow-up, and account for the correlation of the measures in each study as well as the correlation of the repeated measures on the same individual.^21^ Both the intercept (at the study and individual level) and slope (at the individual level) were fitted as random effects with an unstructured covariance matrix at the individual level, allowing for the consideration of study-specific and individual differences in cognitive performance at baseline and individual differences in rate of cognitive decline in model coefficient estimation. Age was used as the time scale and analyses were centred at age 60 years. Initial models for memory and fluency including effects of sex, sex by age, age, age^2^, age^3^, ethnicity, birth cohort, and practice were fit to produce summary measures of cognitive performance and decline. Covariates by practice effect interactions were examined and retained if α was less than 0·05 based on the Wald test; the initial model for fluency additionally included practice effect by sex interaction. The analyses were then adjusted for birth cohort, with models including all covariates in the initial model and higher-order interactions of sex by age, birth cohort by age, and sex by birth cohort by age using backwards selection with α of less than 0·05 based on the Wald test. In addition to covariates included in the initial model, the final birth-cohort adjusted model included birth cohort by age^3^ and lower-order interactions for memory, and birth cohort by sex by age^2^ and lower-order interactions for fluency.

First, we examined the effect of education on differences between sexes in cognitive performance and decline by adding level of education (four categories) and education by age into the initial and birth-cohort adjusted models, as well as education by age^2^ and by age^3^ when α was less than 0·05. We then examined whether the associations between sex and cognitive performance and decline differed by level of education by adding sex by education and sex by education by age interactions to the birth-cohort adjusted models, and reported p values based on the Wald test for these terms. Education was treated as an ordinal variable in this analysis to improve statistical power. Finally, sex differences in cognitive performance and decline were analysed separately in participants with low or high education level.

To facilitate interpretation of results, differences between sexes in cognitive performance were estimated at ages 50, 60, and 70 years. Difference between sexes in cognitive decline over 13 years (the maximum follow-up period for ELSA; mean follow-up period for Whitehall II) from age 60 years was based on predicted values for each birth cohort. We used the Wald test to determine p values for change in differences between sexes in cognitive performance and decline as a function of birth cohort. We did four sets of sensitivity analyses: first, we assessed effects separately in each study; second, we excluded participants with dementia (ascertained in ELSA using participant or proxy report^19^ and in Whitehall II using linkage to electronic health records);^20^ third, we restricted the period of follow-up to the same period (2002–15) in both studies; and fourth, we used multiple imputation (n=20) rather than single imputation for missing data on level of education in models adjusted for education. All analyses were done in Stata 15 and a two-sided p value of less than 0·05 was considered significant.

### Role of the funding source

The funders of the study had no role in study design, data collection, data analysis, data interpretation, or writing of the report. All authors had full access to all the data in the study and had final responsibility for the decision to submit for publication.

## Results

Of 11 391 core participants in wave one of ELSA (2002), 2883 (25·31%) were born before 1930, 96 (0·84%) had missing cognitive data, and 16 (0·14%) had missing covariate data; therefore 8396 participants (73·71%) from ELSA were included in our analyses. Of 10 308 participants in Whitehall II, 306 (2·97%) died, 880 (8·54%) withdrew before the first cognitive wave, and 1594 (15·46%) had missing cognitive data; therefore 7528 (73·03%) participants were included in our analyses. The pooled sample comprised 15 924 participants (appendix p 1).

The ELSA study included almost equal numbers of men and women (3906 [46·52%] of 8396 participants were male), whereas participants in the Whitehall II study were mostly male (5295 [70·34%] of 7528 participants; appendix p 3). In both studies, education level increased in participants born in later birth cohorts (p_trend_<0·0001). For 703 ELSA participants (8·37%) and 370 Whitehall II participants (4·91%), education was imputed using single imputation. Whitehall II participants were more likely than ELSA participants to have high education level (p<0·0001). Men were more likely than women to have high education level (pooled data; 4229 [45·96%] of 9201 men and 1614 [24·01%] of 6723 women; p<0·0001) across all birth cohorts (p<0·0001 in each birth cohort). Education level increased with each successive birth cohort (p<0·0001). In the pooled data, men had more years of follow-up (mean 11·0 years [SD 6·2]) than women (9·6 years [5·7]); p<0·0001), because there was a greater proportion of men in Whitehall II in which the follow-up was longer than in ELSA (12·9 years [SD 6·3] mean follow-up in Whitehall II *vs* 8·1 years [4·8] in ELSA; p<0·0001).

Women had better memory scores than men (mean difference [male scores minus female scores] –0·10 SDs [95% CI –0·15 to –0·05] at age 50 years, –0·14 SDs [–0·18 to –0·11] at 60 years, and –0·19 SDs [–0·22 to –0·16] at 70 years) in analyses adjusted for age, ethnicity, birth cohort, and practice effect. After adjustment for level of education, these differences were even larger (–0·17 SDs [–0·21 to –0·13] at age 50 years, –0·22 SDs [–0·25 to –0·20] at 60 years, and –0·28 SDs [–0·31 to –0·25] at 70 years).

**Figure 1:**
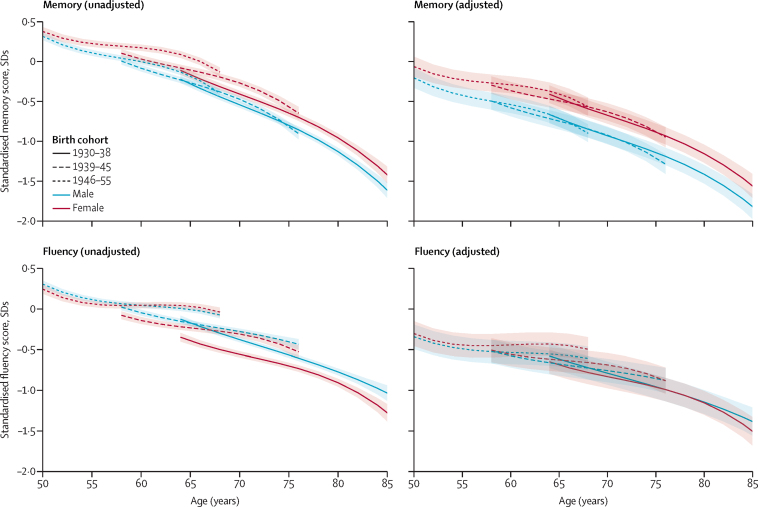
Cognitive performance trajectories before and after adjustment for education Base models adjusted for ethnicity, practice effect, and interactions with age. Results are shown for ethnicity (white) and practice effect (no practice effect) reference categories. Model adjusted for education, and interactions with age is shown for the education reference category (no qualifications).

The higher memory scores in women were evident in each birth cohort, before and after adjustment for level of education (table 1, figure 1). At age 70 years, estimated sex differences in memory performance were larger in participants born in later birth cohorts (p=0·0004 for difference across birth cohorts). This pattern persisted after adjustment for education (p=0·031); the female advantage in the 1930–38 birth cohort was smaller (–0·26 SDs [95% CI –0·30 to –0·21]) than in the 1946–55 birth cohort (–0·36 SDs [–0·42 to –0·29]). Better memory performance in women at age 50 years was seen in the 1946–55 birth cohort (no data were available in earlier birth cohorts for age 50 years) and in all three birth cohorts at age 60 years and age 70 years, irrespective of adjustment for education.

**Table 1:** Role of education in sex differences in cognitive performance, stratified by birth cohort

		**Age 50 years**		**Age 60 years**		**Age 70 years**	
		Base model	Base model adjusted for education	Base model	Base model adjusted for education	Base model	Base model adjusted for education
**Memory**							
Birth cohort							
	1930–38	..	..	−0·10 (−0·16 to −0·03)	−0·25 (−0·32 to −0·19)	−0·13 (−0·18 to −0·09)	−0·26 (−0·30 to −0·21)
	1939–45	..	..	−0·12 (−0·16 to −0·07)	−0·21 (−0·26 to −0·17)	−0·20 (−0·26 to −0·15)	−0·29 (−0·34 to −0·24)
	1946–55	−0·06 (−0·12 to −0·01)	−0·14 (−0·20 to −0·09)	−0·17 (−0·22 to −0·13)	−0·25 (−0·29 to −0·21)	−0·29 (−0·35 to −0·23)	−0·36 (−0·42 to −0·29)
p value for sex difference by birth cohort		..	..	0·070	0·47	0·0004	0·031
**Fluency**							
Birth cohort							
	1930–38	..	..	0·19 (0·08 to 0·31)	0·03 (−0·09 to 0·15)	0·18 (0·13 to 0·23)	0·04 (−0·01 to 0·09)
	1939–45	..	..	0·10 (0·04 to 0·15)	−0·02 (−0·07 to 0·03)	0·03 (−0·02 to 0·08)	−0·07 (−0·12 to −0·02)
	1946–55	0·06 (−0·01 to 0·13)	−0·04 (−0·11 to 0·03)	0·00 (−0·05 to 0·05)	−0·09 (−0·14 to −0·05)	−0·01 (−0·11 to 0·08)	−0·08 (−0·18 to 0·01)
p value for sex difference by birth cohort		..	..	0·0018	0·034	<0·0001	0·0015

Data are mean difference between sexes in SDs (95% CI), or p values. Positive SD values indicate men had better scores; negative values indicate women had better scores. Base models included sex, sex by age, age^2^, age^3^, birth cohort, sex by birth cohort, birth cohort by age, sex by birth cohort by age, ethnicity, and practice effect. Memory models additionally included birth cohort by age^3^ and lower-order interactions. Fluency models additionally included practice effect by sex and birth cohort by sex by age^2^ and lower-order interactions.

**Figure 2:**
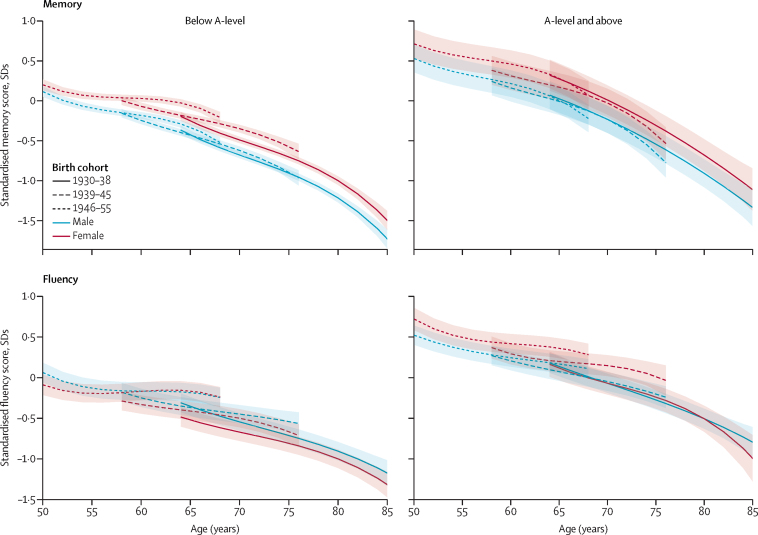
Cognitive performance trajectories stratified by education Adjusted for ethnicity, practice effect, and interactions with age. Results are shown for ethnicity (white) and practice effect (no practice effect) reference categories. Low education was defined as qualifications below A-level; high education was A-level qualifications or higher.

Assessment of sex differences in memory scores in analyses stratified by education showed robust evidence of higher scores in women (table 2, figure 2), with a greater difference at age 70 years than at age 50 years in both low and high education level groups (p<0·0001 for low education group; p=0·044 for high education group). The general pattern of results was similar in both education groups. At age 50 years, the female advantage in memory scores was greater in those with high education (–0**·**18 SDs [95% CI –0·27 to –0·10]) than in those with low education (–0·09 SDs [–0**·**16 to –0**·**01]) in the 1946–55 birth cohort (table 2). In the low education group, the female advantage in memory scores was progressively greater in the later birth cohorts at age 70 years (p=0·0071; sex difference –0·19 SDs [–0·24 to –0·14] in the 1930–38 birth cohort compared with –0·34 SDs [–0·43 to –0·25] in the 1946–55 birth cohort).

**Table 2:** Sex differences in cognitive performance, stratified by birth cohort and level of education

			**Age 50 years**	**Age 60 years**	**Age 70 years**
**Memory**					
Low education					
	Birth cohort				
		1930–38	..	−0·16 (−0·24 to −0·08)	−0·19 (−0·24 to −0·14)
		1939–45	..	−0·18 (−0·24 to −0·12)	−0·27 (−0·34 to −0·21)
		1946–55	−0·09 (−0·16 to −0·01)	−0·21 (−0·27 to −0·16)	−0·34 (−0·43 to −0·25)
	p value for sex difference by birth cohort		..	0·49	0·0071
High education					
	Birth cohort				
		1930–38	..	−0·26 (−0·40 to −0·12)	−0·24 (−0·34 to −0·15)
		1939–45	..	−0·15 (−0·23 to −0·07)	−0·21 (−0·29 to −0·12)
		1946–55	−0·18 (−0·27 to −0·10)	−0·24 (−0·31 to −0·18)	−0·31 (−0·40 to −0·22)
	p value for sex difference by birth cohort		..	0·17	0·28
**Fluency**					
Low education					
	Birth cohort				
		1930–38	..	0·20 (0·05 to 0·36)	0·13 (0·07 to 0·18)
		1939–45	..	0·08 (0·01 to 0·15)	0·05 (−0·02 to 0·12)
		1946–55	0·15 (0·05 to 0·25)	0·00 (−0·06 to 0·06)	0·02 (−0·11 to 0·15)
	p value for sex difference by birth cohort		..	0·028	0·12
High education					
	Birth cohort				
		1930–38	..	−0·05 (−0·26 to 0·15)	0·00 (−0·11 to 0·11)
		1939–45	..	−0·09 (−0·18 to 0·01)	−0·20 (−0·29 to −0·11)
		1946–55	−0·20 (−0·31 to −0·09)	−0·17 (−0·24 to −0·10)	−0·13 (−0·27 to 0·01)
	p value for sex difference by birth cohort		..	0·27	0·023

Data are mean difference between sexes in SDs (95% CI), or p values. Positive SD values indicate men had better scores; negative values indicate women had better scores. Low education was defined as qualifications below A-level; high education was A-level qualifications or higher. Base models included sex, sex by age, age^2^, age^3^, birth cohort, sex by birth cohort, birth cohort by age, sex by birth cohort by age, ethnicity, and practice effect. Memory models additionally included birth cohort by age^3^ and lower-order interactions. Fluency models additionally included practice effect by sex and birth cohort by sex by age^2^ and lower-order interactions.

Men had higher fluency scores than women (mean difference 0·07 SDs [95% CI 0·00 to 0·13] at age 50 years, 0·07 SDs [0·04 to 0·11] at 60 years, and 0·06 SDs [0·03 to 0·09] at 70 years) in analyses accounting for age, ethnicity, birth cohort, practice effect, and practice effect by sex interaction. This difference was attenuated after adjustment for education (–0·04 SDs [–0·11 to 0·02] at age 50 years, –0·04 SDs [–0·07 to –0·01] at 60 years, and –0·04 SDs [–0·07 to –0·01] at 70 years).

The difference in fluency scores was smaller in later birth cohorts (p≤0·040 for all cohorts, table 1, figure 1) compared with the overall scores, and was further attenuated (eg, at age 50 years in the 1946–55 birth cohort) or reversed (eg, at age 60 years in the 1946–55 birth cohort) after adjustment for education. Analyses stratified by education (table 2, figure 2) showed that men had higher fluency scores only in the low education group (p<0·0001 for sex by education interaction). In the high education group, there was evidence of higher fluency scores in women, particularly for those in the 1946–55 birth cohort at age 50 years (–0·20 SDs [–0·31 to –0·09]) and at 60 years (–0·17 SDs [–0·24 to –0·10]).

The mean 13-year change in memory scores was –0·76 SDs (95% CI –0·85 to –0·71) in men and –0·69 SDs (–0·75 to –0·63) in women (p=0·0001; adjusted for age, ethnicity, birth cohort, education, and practice effect). 13-year memory decline was slower in women than in men (sex difference in the 1939–45 birth cohort –0·12 SDs [–0·18 to –0·05] and in the 1946–55 birth cohort –0·15 SDs [–0·20 to –0·09], with no difference in the 1930–38 birth cohort; table 3). Adjustment for education did not affect these estimates.

**Table 3:** Role of education in sex differences in 13-year cognitive decline, stratified by birth cohort

		**Base model**	**Base model adjusted for education**
**Memory**			
Birth cohort			
	1930–38	−0·05 (−0·11 to 0·01)	0·00 (−0·07 to 0·06)
	1939–45	−0·12 (−0·18 to −0·05)	−0·10 (−0·16 to −0·04)
	1946–55	−0·15 (−0·20 to −0·09)	−0·14 (−0·20 to −0·08)
p value for sex difference by birth cohort		0·068	0·0059
**Fluency**			
Birth cohort			
	1930–38	−0·05 (−0·17 to 0·08)	−0·01 (−0·13 to 0·11)
	1939–45	−0·05 (−0·12 to 0·01)	−0·03 (−0·10 to 0·03)
	1946–55	0·06 (−0·11 to 0·24)	0·10 (−0·08 to 0·27)
p value for sex difference by birth cohort		0·48	0·41

Data are mean difference between sexes in SDs (95% CI), or p values. Positive SD values indicate slower cognitive decline in men; negative values indicate slower cognitive decline in women. Results are shown for the reference category: participants aged 60 years. Base models included sex, sex by age, age^2^, age^3^, birth cohort, sex by birth cohort, birth cohort by age, sex by birth cohort by age, ethnicity, and practice effect. Memory models additionally included birth cohort by age^3^ and lower-order interactions. Fluency models additionally included practice effect by sex and birth cohort by sex by age^2^ and lower-order interactions.

The mean 13-year change in fluency scores was similar in men (–0·41 SDs [95% CI –0·46 to –0·36]) and women (–0·40 SDs [–0·45 to –0·34]; p=0·61; adjusted for age, ethnicity, birth cohort, education, practice effect, and practice effect by sex). This finding was consistent in analyses within each birth cohort (table 3), even after adjustment for education.

For memory decline, there was no evidence that sex differences in decline varied by level of education (p=0·081 for sex by education by age interaction; table 4). Women experienced slower decline in memory compared with men, both in the low and high education groups. For decline in fluency, the interaction term between sex, education, and age suggested differences in patterns in the low and high education groups (p=0·0001). This was due to the 1939–45 birth cohort where there was no sex difference in 13-year decline in fluency scores (sex difference 0·00 SDs [95% CI –0·08 to 0·09]) in the low education group and a slower 13-year decline in women than in men (sex difference –0·13 SDs [–0·24 to –0·03]) in the high education group.

**Table 4:** Sex differences in 13-year cognitive decline, stratified by birth cohort and level of education

			**Base model**
**Memory**			
Low education			
	Birth cohort		
		1930–38	−0·04 (−0·11 to 0·04)
		1939–45	−0·12 (−0·21 to −0·04)
		1946–55	−0·17 (−0·24 to −0·09)
	p value for sex difference by birth cohort		0·064
High education			
	Birth cohort		
		1930–38	0·02 (−0·11 to 0·14)
		1939–45	−0·07 (−0·17 to 0·02)
		1946–55	−0·08 (−0·16 to −0·00)
	p value for sex difference by birth cohort		0·41
**Fluency**			
Low education			
	Birth cohort		
		1930–38	−0·10 (−0·25 to 0·06)
		1939–45	0·00 (−0·08 to 0·09)
		1946–55	0·08 (−0·16 to 0·32)
	p value for sex difference by birth cohort		0·29
High education			
	Birth cohort		
		1930–38	0·03 (−0·18 to 0·24)
		1939–45	−0·13 (−0·24 to −0·03)
		1946–55	0·16 (−0·11 to 0·44)
	p value for sex difference by birth cohort		0·11

Data are mean difference between sexes in SDs (95% CI), or p values. Positive SD values indicate slower cognitive decline in men; negative values indicate slower cognitive decline in women. Low education was defined as qualifications below A-level; high education was A-level qualifications or higher. Results are shown for the reference category: participants aged 60 years. Base models included sex, sex by age, age^2^, age^3^, birth cohort, sex by birth cohort, birth cohort by age, sex by birth cohort by age, ethnicity, and practice effect. Memory models additionally included birth cohort by age^3^ and lower-order interactions. Fluency models additionally included practice effect by sex and birth cohort by sex by age^2^ and lower-order interactions.

The analyses done separately in the ELSA and Whitehall II studies showed results broadly similar to those in the pooled analyses for cognitive performance (appendix pp 4–5) and decline (appendix p 6), although differences in level of education in these studies were reflected in the estimates. In the ELSA study, results were similar to those in the low education group in the pooled analyses and in the Whitehall II study, results were similar to those in the high education group in the pooled analyses. Neither omitting respondents with dementia (appendix pp 7–10), limiting years of follow-up to 2002–15 (appendix pp 11–14), nor using multiple imputation rather than single imputation for missing data on level of education (appendix pp 15–16) substantially impacted the results.

## Discussion

In this analysis of 15 924 men and women born between 1930 and 1955 from two prospective cohort studies, with longitudinal data on memory and fluency spanning up to 19 years, there was no evidence of a cognitive disadvantage in women after accounting for level of education. On the contrary, women had better scores than men on the memory test, and this difference was more marked at older ages and in women born more recently. For fluency, there was evidence of an effect of education and birth cohort. Women in the high education group and in the latest birth cohort had better fluency scores than men; men in the low education group and in the earliest birth cohort had better fluency scores than women. Women experienced slower rates of memory decline than men, although there was no strong evidence of sex differences in fluency decline. These findings suggest a role of education and secular changes in education level in successive birth cohorts in determining cognitive performance in women.

A major strength of our study is its large sample size compared to previous longitudinal studies on the role of education in sex differences in cognition,^11^, ^12^, ^13^ the largest of which comprised 2225 participants.^12^ Pooling data from two large studies allowed sufficient statistical power to examine our hypothesis. Differences in target population and study design—including between-study differences due to written versus oral cognitive tests—were addressed by standardising the cognitive measures within each study, including a random effect for study, and by doing sensitivity analyses separately in each study. Our analytical approach considered both the role of birth cohort and level of education in sex differences in cognitive trajectories, allowing secular changes in education level in the mid-20th century to be considered. Another strength is the long follow-up period, which allowed us to examine cognitive trajectories from midlife to older ages.

Our study has some limitations. The ability of linear mixed models to handle incomplete data is dependent on the assumption that data are missing at random, which might not be correct in this study.^15^ The effect of attrition on memory scores in the ELSA study was previously examined using joint models and estimates were similar to those using linear mixed models.^15^ A paper from the Whitehall II study also found a similar association of socioeconomic factors with cognitive performance and decline when estimates from mixed models were compared with simulations with a missing-not-at-random assumption.^22^ Differences in mean follow-up between men and women in each cohort were small compared with the overall mean follow-up duration (1·3 years difference in Whitehall II and 0·5 years in ELSA, representing at most 10% of the mean follow-up duration of these studies). Thus, the effect of attrition on our findings is likely to be small. Participants in the ELSA and Whitehall II studies are mostly white, and the extent to which these results are generalisable to other racial and ethnic groups is unknown. However, the ethnic composition of both studies reflects the population in England for the birth years included in the analyses.^23^ Sex in both studies was measured as declared in administrative documents rather than by gender identity. The effect of education on cognition is likely to arise through expectations of gender roles rather than effects of biological sex, but absence of data on gender leads us to refer to sex rather than gender differences. Detailed analysis of education using years of schooling, or more categories than our four-category measure, is likely to affect findings, perhaps strengthening the attenuation in sex differences after adjustment for level of education. To enable education-stratified analyses, we used single imputation for missing data on education. The use of single imputation rather than multiple imputation can underestimate SEs, but in this case it did not appear to substantially impact our findings as a relatively small proportion of participants had missing data (1073 [6·74%] of 15 924). Finally, we could examine only two cognitive tests as these were the tests available in both studies. The extent to which our findings extend to other cognitive domains remains to be examined.

Our results are consistent with previous studies which have found domain-specific differences between sexes in cognitive performance and decline.^12^, ^24^ As in previous studies,^16^, ^17^, ^18^ women consistently had better memory scores than men. The underlying mechanisms of observed differences in memory between sexes are not well understood,^25^ although the neuroprotective effect of oestradiol has been identified as a possible explanation.^26^ However, consistent with de Frias and colleagues,^26^ our results do not support this hypothesis, as we observed a smaller difference between sexes at perimenopausal and premenopausal age (50 years) compared with menopausal age groups (60 and 70 years).

Previous studies of differences between sexes in semantic fluency scores have had inconsistent findings, with some showing male^12^ or female^27^ advantages, and others showing no sex differences.^13^, ^17^, ^18^ The findings of our analysis offer one possible explanation for these mixed results, as we found male advantages in fluency scores among those in earlier birth cohorts and lesser sex differences among those in later birth cohorts. Furthermore, stratification by level of education showed either no sex difference or a female advantage in the high education group, and a male advantage in the low education group. These results suggest that differences in fluency between sexes depend on birth cohort and level of education.

Some studies found that women experienced slower cognitive decline than men across multiple cognitive domains;^27^, ^28^ however, others did not find a difference between sexes.^24^, ^26^, ^27^ Our data show a slower rate of memory decline in women compared with men, but there was little evidence of a difference between sexes in fluency decline. One previous study reported that level of education did not affect sex differences in memory and fluency performance and decline in a sample size of 2225 adults, aged 31 years and older over 27 years of follow-up.^12^ Another study of 368 adults aged older than 70 years at baseline and followed up over 13 years came to the same conclusion.^13^ Our study is based on a larger sample size and has the advantage of explicit consideration of birth cohort effects to reflect the changes in access to education, particularly in women. We found that education had an important role in determining performance, but not decline, on memory and fluency tests.

Our findings need to be considered within the lifecourse perspective of cognitive ageing.^29^ Midlife cognitive performance reflects peak cognition, which is influenced by early life, educational opportunities, and a range of environmental exposures.^30^ In turn, cognitive performance in adulthood is important for late-life outcomes such as cognitive impairment and Alzheimer's disease and related dementias, as higher cognitive performance delays the time at which individuals reach the threshold for clinically diagnosable impairment.^8^ Education is consistently associated with higher cognitive performance but not with cognitive decline,^8^ supporting the contribution of education to cognitive reserve, whereby the protective association of education for Alzheimer's disease and related dementias is due to the effect of education on peak performance rather than on the rate of cognitive decline.^22^ We found that education had a similar role in sex differences in cognition, suggesting that the historical reduced access to higher education due to gendered expectations of educational attainment could have contributed to higher rates of Alzheimer's disease and related dementias in women.

Identification of factors that affect adult cognitive performance or decline is important to develop interventions that offer protection against adverse cognitive outcomes at older ages.^31^ Continuing secular increases in access to education among women and the subsequent impact on cognitive function could change late-life cognitive outcomes for women in the future. A commonly cited meta-analysis that found a 50% increased risk of Alzheimer's disease and related dementias in women compared with men was primarily based on studies of people born in the 1920s and earlier,^4^ a period during which differences between sexes in access to education were particularly large.^32^ Given the increase in mean education level over the past century and the reduction in sex differences in access to education in some but not all countries,^32^, ^33^ it remains to be seen whether the increased risk of Alzheimer's disease and related dementias among women will persist in the future. Our finding that women in more recent birth cohorts outperform men in memory and fluency tests and have slower memory decline suggests that the sex differences in Alzheimer's disease and related dementias risk might be attenuated in later birth cohorts, due in part to a secular decrease in sex disparities in access to education.

There are other potential explanations for sex differences in Alzheimer's disease and related dementias and the apparent discrepancy between the advantage of females in midlife memory performance and decline and their increased risk of developing these diseases. Indeed, there is evidence that the female advantage in memory performance is attenuated among patients with dementia, suggesting that sex differences in cognitive trajectory among people with dementia might differ from those of the cognitively healthy.^34^ In addition, dementia is multifactorial; there are multiple risk factors (eg, hormonal, behavioural, cardiovascular, metabolic), and sociodemographic disparities in the use of health-care services and study participation must also be considered, as the diagnosis of dementia is not straightforward. As such, the extent to which population change in sex differences in education will be reflected in the frequency of dementia remains to be elucidated and is an area for future research. Replication of these analyses in future studies including a larger range of birth cohorts would provide further insight into the role of education across the lifecourse.

Sex differences in cognitive outcomes in older adults have often been examined simply as the excess risk of Alzheimer's disease and related dementias in women. Our analysis by level of education and birth cohort shows sex differences in cognitive performance are dynamic, whereby women in later birth cohorts increasingly have better memory scores and the deficit in fluency has progressively been attenuated such that women have higher fluency scores than men in the high education group. There was also evidence of slower decline in memory in women than in men. These findings highlight the importance of education as a contributing factor for sex differences in cognition, accentuate the necessity of considering sex-specific effects when evaluating modifiable factors for cognitive outcomes, and emphasise the importance of equitable access to education for health.

## Data sharing

The Whitehall II data cannot be shared publicly because of constraints dictated by the study's ethics approval and institutional review board restrictions. The Whitehall II data are available for sharing within the scientific community. Researchers can apply for data access at: https://www.ucl.ac.uk/epidemiology-health-care/research/epidemiology-and-public-health/research/whitehall-ii/data-sharing. The ELSA data are freely available to researchers through the UK Data Service at: https://beta.ukdataservice.ac.uk/datacatalogue/series/series?id=200011.
