# Supplementary material for: Sex differences and the role of education in cognitive ageing: analysis of two UK-based prospective cohort studies
Source: Lancet Public Health. 2021 Jan 28;6(2):e106–15. doi: 10.1016/S2468-2667(20)30258-9 (PMC8141610; doi:10.1016/S2468-2667(20)30258-9)
Supplement: Supplementary appendix [file mmc1.pdf]

THE LANCET
Public Health

Supplementary appendix

This appendix formed part of the original submission and has been peer reviewed. We post it as supplied by the authors.

Supplement to: Bloomberg M, Dugravot A, Dumurgier J, et al. Sex differences and the role of education in cognitive ageing: analysis of two UK-based prospective cohort studies. *Lancet Public Health* 2021; **6:** e106–15.

Figure S1. Flow chart.

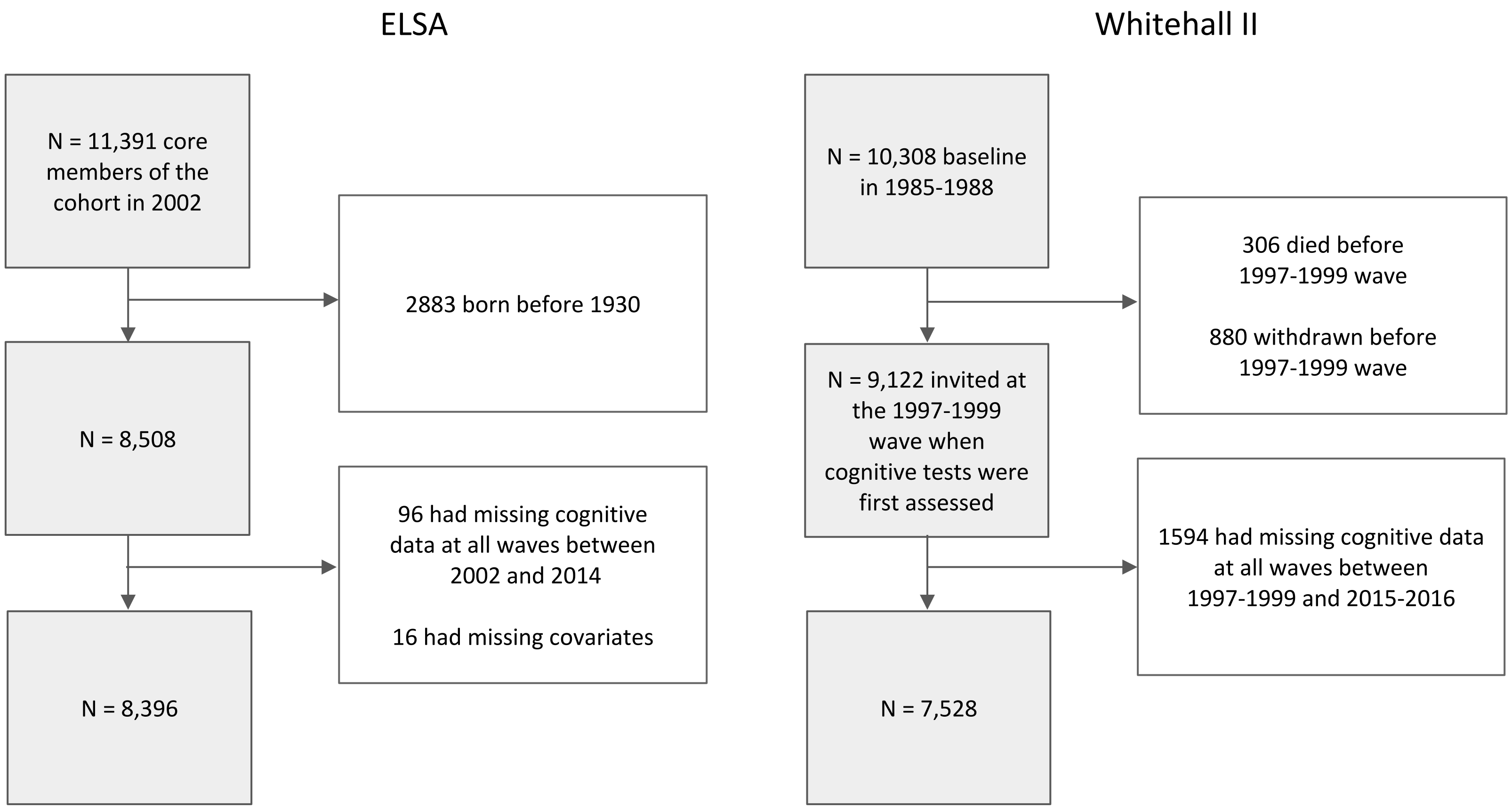

Table S1. Education categories available in Whitehall II and ELSA.

<table>
<tr><th>Whitehall II</th><th>ELSA</th><th>Education-adjusted models[a]</th><th>Education-stratified models[b]</th></tr>
<tr><td>No qualification</td><td>No qualification</td><td>Less than secondary</td><td rowspan="2">Low education</td></tr>
<tr><td>O-level</td><td>O-level</td><td>O-level</td></tr>
<tr><td rowspan="2">A-level</td><td>A-level</td><td rowspan="2">A-level</td><td rowspan="4">High education</td></tr>
<tr><td>Higher education below degree level</td></tr>
<tr><td>BA/BSc</td><td rowspan="2">University degree and above</td><td rowspan="2">University degree and above</td></tr>
<tr><td>Higher degree</td></tr>
</table>

[a]Models adjusted for education include 4-class education variable as a categorical covariate. Models adjusted for education by sex interactions include education as a continuous variable to increase statistical power.
[b]Models stratified by education use this specification for high and low education categories.

Table S2. Characteristics of participants included in the analyses from ELSA and the Whitehall II study.

	1930-1938			1939-1945			1946-1955			P trend Men	P trend Women
	Men	Women	P-value	Men	Women	P-value	Men	Women	P-value		
**ELSA**	**N = 1377**	**N = 1525**		**N = 1153**	**N = 1338**		**N = 1376**	**N = 1627**			
Baseline Age, M(SD)	67·9 (2·6)	68·1 (2·6)	0·16	60·1 (2·1)	60·0 (2·1)	0·18	53·5 (2·0)	53·4 (2·0)	0·40	<0·0001	<0·0001
Ethnicity, N (%)											
White	1322 (96·0)	1487 (97·5)	0·021	1116 (96·8)	1308 (97·8)	0·14	1325 (96·3)	1570 (96·5)	0·76	0·69	0·080
Non-white	55 (4·0)	38 (2·5)		37 (3·2)	30 (2·2)		51 (3·7)	57 (3·5)			
Education, N (%)											
Below A-level	1133 (82·3)	1401 (91·9)	<0·0001	863 (74·8)	1130 (84·5)	<0·0001	928 (67·4)	1299 (79·8)	<0·0001	<0·0001	<0·0001
A-level and above	244 (17·7)	124 (8·1)		290 (25·2)	208 (15·5)		448 (32·6)	328 (20·2)			
**Whitehall II**	**N = 1599**	**N = 793**		**N = 1704**	**N = 722**		**N = 1992**	**N = 718**			
Baseline Age, M(SD)	64·2 (3·6)	64·8 (4·3)	0·00030	56·9 (3·9)	57·2 (3·9)	0·12	51·0 (3·9)	50·9 (3·9)	0·49	<0·0001	<0·0001
Ethnicity, N (%)											
White	1455 (91·0)	693 (87·4)	0·0061	1587 (93·1)	590 (81·7)	<0·0001	1912 (96·0)	638 (88·9)	<0·0001	<0·0001	0·48
Non-white	144 (9·0)	100 (12·6)		117 (6·9)	132 (18·3)		80 (4·0)	80 (11·1)			
Education, N (%)											
Below A-level	777 (48·6)	588 (74·1)	<0·0001	661 (38·8)	410 (56·8)	<0·0001	610 (30·6)	281 (39·1)	<0·0001	<0·0001	<0·0001
A-level and above	822 (51·4)	205 (25·9)		1043 (61·2)	312 (43·2)		1382 (69·4)	437 (60·9)			
**ELSA & Whitehall II**	**N = 2976**	**N = 2318**		**N = 2857**	**N = 2060**		**N = 3368**	**N = 2345**			
Baseline Age, M(SD)	65·9 (3·7)	66·9 (3·6)	<0·0001	58·2 (3·6)	59·0 (3·2)	<0·0001	52·0 (3·5)	52·6 (2·9)	<0·0001	<0·0001	<0·0001
Ethnicity, N (%)											
White	2777 (93·3)	2180 (94·0)	0·28	2703 (94·6)	1898 (92·1)	0·00050	3237 (96·1)	2208 (94·2)	0·00060	<0·0001	0·87
Non-white	199 (6·7)	138 (6·0)		154 (5·4)	162 (7·9)		131 (3·9)	137 (5·8)			
Education, N (%)											
Below A-level	1910 (64·2)	1989 (85·8)	<0·0001	1524 (53·3)	1540 (74·8)	<0·0001	1538 (45·7)	1580 (67·4)	<0·0001	<0·0001	<0·0001
A-level and above	1066 (35·8)	329 (14·2)		1333 (46·7)	520 (25·2)		1830 (54·3)	765 (32·6)			

Table S3. Role of education in sex differences in cognitive performance in the Whitehall II study.

	Age 50 years		Age 60 years		Age 70 years	
	Base Model[a]	Basic Model[a] + Education	Basic Model[a]	Basic Model[a] + Education	Basic Model[a]	Basic Model[a] + Education
	**Sex difference (95% CI)**	**Sex difference (95% CI)**	**Sex difference (95% CI)**	**Sex difference (95% CI)**	**Sex difference (95% CI)**	**Sex difference (95% CI)**
**Memory**						
Birth cohort						
1930-1938	No data	No data	-0·06 (-0·16, 0·03)	-0·20 (-0·30, -0·11)	-0·02 (-0·08, 0·05)	-0·12 (-0·19, -0·06)
1939-1945	No data	No data	-0·06 (-0·14, 0·01)	-0·14 (-0·21, -0·07)	-0·06 (-0·13, 0·01)	-0·13 (-0·20, -0·06)
1946-1955	-0·17 (-0·26, -0·09)	-0·21 (-0·29, -0·12)	-0·18 (-0·24, -0·11)	-0·21 (-0·28, -0·15)	-0·18 (-0·27, -0·10)	-0·22 (-0·30, -0·14)
P sex difference by birth cohort			*0·031*	*0·27*	*0·0078*	*0·14*
**Fluency**						
Birth cohort						
1930-1938	No data	No data	0·38 (0·29, 0·47)	0·17 (0·09, 0·25)	0·33 (0·27, 0·40)	0·14 (0·08, 0·21)
1939-1945	No data	No data	0·11 (0·03, 0·18)	-0·02 (-0·09, 0·04)	0·06 (-0·02, 0·13)	-0·06 (-0·13, 0·01)
1946-1955	-0·10 (-0·18, -0·01)	-0·16 (-0·24, -0·09)	-0·11 (-0·17, -0·04)	-0·17 (-0·23, -0·10)	-0·11 (-0·19, -0·03)	-0·17 (-0·25, -0·10)
P sex difference by birth cohort			*<0·0001*	*<0·0001*	*<0·0001*	*<0·0001*

[a]Adjusted for ethnicity, practice effect, interactions with age.
Positive value indicates male advantage in performance.

Table S4. Role of education in sex differences in cognitive performance in ELSA.

	Age 50 years		Age 60 years		Age 70 years	
	Basic Model[a]	Basic Model[a] + Education	Basic Model[a]	Basic Model[a] + Education	Basic Model[a]	Basic Model[a] + Education
	**Sex difference (95% CI)**	**Sex difference (95% CI)**	**Sex difference (95% CI)**	**Sex difference (95% CI)**	**Sex difference (95% CI)**	**Sex difference (95% CI)**
**Memory**						
Birth cohort						
1930-1938	No data	No data	No data	No data	-0·22 (-0·29, -0·16)	-0·35 (-0·41, -0·29)
1939-1945	No data	No data	-0·18 (-0·25, -0·11)	-0·27 (-0·34, -0·21)	-0·25 (-0·32, -0·17)	-0·36 (-0·43, -0·28)
1946-1955	-0·09 (-0·17, -0·01)	-0·17 (-0·25, -0·09)	-0·16 (-0·21, -0·10)	-0·25 (-0·30, -0·20)	No data	No data
P sex difference by birth cohort			*0·69*	*0·47*	*0·90*	*0·83*
**Fluency**						
Birth cohort						
1930-1938	No data	No data	No data	No data	0·11 (0·04, 0·17)	0·00 (-0·07, 0·06)
1939-1945	No data	No data	0·07 (-0·00, 0·14)	-0·02 (-0·09, 0·05)	0·07 (-0·01, 0·15)	-0·03 (-0·11, 0·05)
1946-1955	0·17 (0·09, 0·25)	0·08 (-0·00, 0·16)	0·09 (0·03, 0·15)	0·00 (-0·06, 0·06)	No data	No data
P sex difference by birth cohort			*0·57*	*0·65*	*0·27*	*0·47*

[a]Adjusted for ethnicity, practice effect, interactions with age.
Positive value indicates male advantage in performance.

Table S5. Role of education in sex differences in 13-year cognitive decline: Analyses stratified by birth cohort and undertaken separately in Whitehall II and ELSA.

	Basic Model[a]	Basic Model[a] + Education
	Sex difference (95% CI)	Sex difference (95% CI)
**Whitehall II**		
**Memory**		
Birth cohort		
1930-1938	0·06 (-0·02, 0·14)	0·10 (0·02, 0·19)
1939-1945	0·00 (-0·07, 0·08)	0·01 (-0·06, 0·09)
1946-1955	-0·01 (-0·08, 0·06)	-0·01 (-0·08, 0·06)
P sex difference by birth cohort	*0·43*	*0·10*
**Fluency**		
Birth cohort		
1930-1938	-0·06 (-0·13, 0·00)	-0·04 (-0·10, 0·03)
1939-1945	-0·06 (-0·12, -0·00)	-0·05 (-0·11, 0·01)
1946-1955	-0·01 (-0·07, 0·05)	-0·01 (-0·06, 0·05)
P sex difference by birth cohort	*0·37*	*0·53*
**ELSA**		
**Memory**		
Birth cohort		
1930-1938	-0·02 (-0·12, 0·08)	-0·04 (-0·14, 0·07)
1939-1945	-0·09 (-0·19, 0·02)	-0·11 (-0·22, -0·01)
1946-1955	-0·09 (-0·18, 0·00)	-0·10 (-0·19, -0·00)
P sex difference by birth cohort	*0·54*	*0·57*
**Fluency**		
Birth cohort		
1930-1938	-0·04 (-0·14, 0·06)	-0·05 (-0·15, 0·05)
1939-1945	0·00 (-0·10, 0·10)	-0·01 (-0·11, 0·09)
1946-1955	-0·10 (-0·19, -0·01)	-0·10 (-0·19, -0·01)
P sex difference by birth cohort	*0·32*	*0·42*

[a]Adjusted for ethnicity, practice effect, interactions with age. Results are shown for the reference category: participants aged 60 years.
Positive value indicates slower cognitive decline in men.

Table S6. Role of education in sex differences in cognitive performance: Analyses stratified by birth cohort and excluding participants with dementia.[a]

	Age 50 years		Age 60 years		Age 70 years	
	Basic Model[b]	Basic Model[b] + Education	Basic Model[b]	Basic Model[b] + Education	Basic Model[b]	Basic Model[b] + Education
**ELSA & Whitehall II**	**Sex difference (95% CI)**	**Sex difference (95% CI)**	**Sex difference (95% CI)**	**Sex difference (95% CI)**	**Sex difference (95% CI)**	**Sex difference (95% CI)**
**Memory**						
Birth cohort						
1930-1938	No data	No data	-0·12 (-0·20, -0·04)	-0·27 (-0·34, -0·19)	-0·14 (-0·19, -0·09)	-0·26 (-0·30, -0·21)
1939-1945	No data	No data	-0·13 (-0·18, -0·07)	-0·22 (-0·27, -0·17)	-0·22 (-0·27, -0·16)	-0·30 (-0·35, -0·25)
1946-1955	-0·07 (-0·13, -0·01)	-0·14 (-0·20, -0·09)	-0·18 (-0·22, -0·14)	-0·25 (-0·29, -0·21)	-0·29 (-0·35, -0·22)	-0·36 (-0·42, -0·29)
P sex difference by birth cohort			*0·22*	*0·46*	*0·0019*	*0·028*
**Fluency**						
Birth cohort						
1930-1938	No data	No data	0·19 (0·07, 0·31)	0·03 (-0·09, 0·15)	0·18 (0·13, 0·24)	0·05 (-0·00, 0·10)
1939-1945	No data	No data	0·09 (0·03, 0·14)	-0·02 (-0·08, 0·03)	0·02 (-0·04, 0·07)	-0·08 (-0·14, -0·03)
1946-1955	0·06 (-0·01, 0·13)	-0·04 (-0·11, 0·03)	0·00 (-0·05, 0·04)	-0·10 (-0·14, -0·05)	-0·02 (-0·11, 0·08)	-0·09 (-0·18, 0·01)
P sex difference by birth cohort			*0·0023*	*0·038*	*<0·0001*	*0·00030*

[a]This analysis is conducted on 15,372 participants free of dementia during the follow-up period (N dementia cases excluded: 434 in Whitehall II and 118 in ELSA).

[b]Basic models include sex, sex by age, age^2, age^3, birth cohort, sex by birth cohort, birth cohort by age, sex by birth cohort by age, ethnicity, and practice effect. Memory models additionally include birth cohort by age^3 and lower-order interactions. Fluency models additionally include practice effect by sex and birth cohort by sex by age^2 and lower-order interactions.

Positive value indicates male advantage in performance.

Table S7. Sex differences in cognitive performance: Analyses stratified by birth cohort and education and excluding participants with dementia.[a]

ELSA & Whitehall II	**Age 50 years** Sex difference (95% CI)	**Age 60 years** Sex difference (95% CI)	**Age 70 years** Sex difference (95% CI)
**Memory**			
**Education: Below A-level**			
1930-1938	No data	-0·18 (-0·27, -0·10)	-0·19 (-0·24, -0·14)
1939-1945	No data	-0·18 (-0·24, -0·12)	-0·29 (-0·35, -0·23)
1946-1955	-0·09 (-0·16, -0·02)	-0·22 (-0·27, -0·16)	-0·34 (-0·43, -0·26)
P sex difference by birth cohort		*0·68*	*0·0042*
**Education: A-level and above**			
1930-1938	No data	-0·23 (-0·38, -0·09)	-0·24 (-0·33, -0·14)
1939-1945	No data	-0·15 (-0·23, -0·06)	-0·20 (-0·28, -0·11)
1946-1955	-0·18 (-0·26, -0·10)	-0·24 (-0·30, -0·18)	-0·30 (-0·39, -0·21)
P sex difference by birth cohort		*0·19*	*0·25*
**Fluency**			
**Education: Below A-level**			
1930-1938	No data	0·23 (0·07, 0·38)	0·13 (0·07, 0·19)
1939-1945	No data	0·08 (0·01, 0·15)	0·03 (-0·03, 0·10)
1946-1955	0·16 (0·06, 0·25)	0·00 (-0·06, 0·06)	0·01 (-0·12, 0·15)
P sex difference by birth cohort		*0·016*	*0·036*
**Education: A-level and above**			
1930-1938	No data	-0·07 (-0·28, 0·14)	-0·01 (-0·12, 0·11)
1939-1945	No data	-0·10 (-0·19, -0·00)	-0·20 (-0·29, -0·11)
1946-1955	-0·20 (-0·30, -0·09)	-0·18 (-0·25, -0·10)	-0·13 (-0·27, 0·01)
P sex difference by birth cohort		*0·34*	*0·028*

[a]This analysis is conducted on 15,372 participants free of dementia during the follow-up period (N dementia cases excluded: 434 in Whitehall II and 118 in ELSA).

Models include sex, sex by age, age^2, age^3, birth cohort, sex by birth cohort, birth cohort by age, sex by birth cohort by age, ethnicity, and practice effect. Memory models additionally include birth cohort by age^3 and lower-order interactions. Fluency models additionally include practice effect by sex and birth cohort by sex by age^2 and lower-order interactions.

Positive value indicates male advantage in performance.

Table S8. Role of education in sex differences in 13-year cognitive decline: Analyses stratified by birth cohort and excluding participants with dementia.[a]

	Basic Model[b]	Basic Model[b] + Education
**ELSA & Whitehall II**	**Sex difference (95% CI)**	**Sex difference (95% CI)**
**Memory**		
Birth cohort		
1930-1938	-0·02 (-0·09, 0·05)	0·01 (-0·05, 0·08)
1939-1945	-0·12 (-0·18, -0·05)	-0·11 (-0·17, -0·05)
1946-1955	-0·14 (-0·20, -0·08)	-0·14 (-0·19, -0·08)
P sex difference by birth cohort	*0·019*	*0·0017*
**Fluency**		
Birth cohort		
1930-1938	-0·04 (-0·16, 0·09)	-0·01 (-0·13, 0·11)
1939-1945	-0·06 (-0·13, 0·00)	-0·05 (-0·11, 0·02)
1946-1955	0·06 (-0·11, 0·23)	0·09 (-0·09, 0·26)
P sex difference by birth cohort	*0·46*	*0·38*

[a]This analysis is conducted on 15,372 participants free of dementia during the follow-up period (N dementia cases excluded: 434 in Whitehall II and 118 in ELSA).
[b]Basic models include sex, sex by age, age^2, age^3, birth cohort, sex by birth cohort, birth cohort by age, sex by birth cohort by age, ethnicity, and practice effect. Memory models additionally include birth cohort by age^3 and lower-order interactions. Fluency models additionally include practice effect by sex and birth cohort by sex by age^2 and lower-order interactions. Results are shown for the reference category: participants aged 60 years.
Positive value indicates slower cognitive decline in men.

Table S9. Sex differences in 13-year cognitive decline: Analyses stratified by birth cohort and education, and excluding participants with dementia.[a]

ELSA & Whitehall II	Sex difference (95% CI)
**Memory**	
**Education: Below A-level**	
1930-1938	-0·01 (-0·09, 0·07)
1939-1945	-0·14 (-0·22, -0·06)
1946-1955	-0·16 (-0·24, -0·09)
P sex difference by birth cohort	*0·012*
**Education: A-level and above**	
1930-1938	-0·01 (-0·14, 0·12)
1939-1945	-0·07 (-0·16, 0·03)
1946-1955	-0·08 (-0·16, -0·00)
P sex difference by birth cohort	*0·64*
**Fluency**	
**Education: Below A-level**	
1930-1938	-0·11 (-0·28, 0·05)
1939-1945	-0·02 (-0·10, 0·06)
1946-1955	0·07 (-0·17, 0·31)
P sex difference by birth cohort	*0·30*
**Education: A-level and above**	
1930-1938	0·05 (-0·16, 0·27)
1939-1945	-0·13 (-0·23, -0·02)
1946-1955	0·16 (-0·11, 0·43)
P sex difference by birth cohort	*0·10*

[a]This analysis is conducted on 15,372 participants free of dementia during the follow-up period (N dementia cases excluded: 434 in Whitehall II and 118 in ELSA).

Models include sex, sex by age, age^2, age^3, birth cohort, sex by birth cohort, birth cohort by age, sex by birth cohort by age, ethnicity, and practice effect. Memory models additionally include birth cohort by age^3 and lower-order interactions. Fluency models additionally include practice effect by sex and birth cohort by sex by age^2 and lower-order interactions. Results are shown for the reference category: participants aged 60 years.

Positive value indicates slower cognitive decline in men.

Table S10. Role of education in sex differences in cognitive performance: Analyses stratified by birth cohort and restricted to follow-up period 2002 to 2015.[a]

	**Age 50 years**		**Age 60 years**		**Age 70 years**	
	Basic Model[b]	Basic Model[b] + Education	Basic Model[b]	Basic Model[b] + Education	Basic Model[b]	Basic Model[b] + Education
**ELSA & Whitehall II**	**Sex difference (95% CI)**	**Sex difference (95% CI)**	**Sex difference (95% CI)**	**Sex difference (95% CI)**	**Sex difference (95% CI)**	**Sex difference (95% CI)**
**Memory**						
Birth cohort						
1930-1938	No data	No data	No data	No data	-0·14 (-0·20, -0·09)	-0·26 (-0·30, -0·21)
1939-1945	No data	No data	-0·14 (-0·19, -0·08)	-0·23 (-0·28, -0·18)	-0·21 (-0·27, -0·16)	-0·30 (-0·35, -0·25)
1946-1955	-0·06 (-0·13, 0·01)	-0·14 (-0·20, -0·07)	-0·17 (-0·22, -0·13)	-0·25 (-0·29, -0·21)	No data	No data
P sex difference by birth cohort			*0·31*	*0·58*	*0·080*	*0·21*
**Fluency**						
Birth cohort						
1930-1938	No data	No data	No data	No data	0·18 (0·13, 0·23)	0·05 (-0·00, 0·10)
1939-1945	No data	No data	0·10 (0·04, 0·16)	0·00 (-0·06, 0·05)	0·04 (-0·02, 0·10)	-0·06 (-0·11, -0·01)
1946-1955	0·12 (0·01, 0·23)	0·03 (-0·08, 0·14)	-0·01 (-0·06, 0·04)	-0·10 (-0·14, -0·05)	No data	No data
P sex difference by birth cohort			*0·0031*	*0·011*	*0·00040*	*0·0041*

[a]N = 15,368.
[b]Basic models include sex, sex by age, age^2, age^3, birth cohort, sex by birth cohort, birth cohort by age, sex by birth cohort by age, ethnicity, and practice effect. Memory models additionally include birth cohort by age^3 and lower-order interactions. Fluency models additionally include practice effect by sex and birth cohort by sex by age^2 and lower-order interactions.
Positive value indicates male advantage in performance.

Table S11. Sex differences in cognitive performance: Analyses stratified by birth cohort and education, and restricted to follow-up period 2002 to 2015.[a]

ELSA & Whitehall II	**Age 50 years** Sex difference (95% CI)	**Age 60 years** Sex difference (95% CI)	**Age 70 years** Sex difference (95% CI)
**Memory**			
**Education: Below A-level**			
1930-1938	No data	No data	-0·20 (-0·26, -0·15)
1939-1945	No data	-0·18 (-0·25, -0·12)	-0·28 (-0·35, -0·21)
1946-1955	-0·10 (-0·18, -0·02)	-0·21 (-0·27, -0·16)	No data
P sex difference by birth cohort		*0·47*	*0·079*
**Education: A-level and above**			
1930-1938	No data	No data	-0·24 (-0·34, -0·13)
1939-1945	No data	-0·18 (-0·28, -0·09)	-0·22 (-0·31, -0·13)
1946-1955	-0·14 (-0·24, -0·04)	-0·24 (-0·30, -0·18)	No data
P sex difference by birth cohort		*0·31*	*0·83*
**Fluency**			
**Education: Below A-level**			
1930-1938	No data	No data	0·12 (0·07, 0·18)
1939-1945	No data	0·09 (0·02, 0·16)	0·06 (-0·01, 0·13)
1946-1955	0·17 (0·02, 0·31)	0·00 (-0·06, 0·06)	No data
P sex difference by birth cohort		*0·051*	*0·18*
**Education: A-level and above**			
1930-1938	No data	No data	0·03 (-0·08, 0·14)
1939-1945	No data	-0·08 (-0·18, 0·02)	-0·20 (-0·29, -0·10)
1946-1955	-0·18 (-0·36, 0·00)	-0·17 (-0·24, -0·09)	No data
P sex difference by birth cohort		*0·18*	*0·0028*

[a]N = 15,368.

Models include sex, sex by age, age^2, age^3, birth cohort, sex by birth cohort, birth cohort by age, sex by birth cohort by age, ethnicity, and practice effect. Memory models additionally include birth cohort by age^3 and lower-order interactions. Fluency models additionally include practice effect by sex and birth cohort by sex by age^2 and lower-order interactions.

Positive value indicates male advantage in performance.

Table S12. Role of education in sex differences in 13-year cognitive decline: Analyses stratified by birth cohort and restricted to follow-up period 2002 to 2015.[a]

	Basic Model[b]	Basic Model[b] + Education
**ELSA & Whitehall II**	**Sex difference (95% CI)**	**Sex difference (95% CI)**
**Memory**		
Birth cohort		
1930-1938	No data	No data
1939-1945	-0·09 (-0·18, -0·01)	-0·09 (-0·17, -0·02)
1946-1955	-0·15 (-0·23, -0·08)	-0·14 (-0·21, -0·07)
P sex difference by birth cohort	*0·28*	*0·33*
**Fluency**		
Birth cohort		
1930-1938	No data	No data
1939-1945	-0·03 (-0·11, 0·04)	-0·02 (-0·10, 0·05)
1946-1955	0·14 (-0·11, 0·39)	0·16 (-0·09, 0·41)
P sex difference by birth cohort	*0·20*	*0·17*

[a]N = 15,368.
[b]Basic models include sex, sex by age, age^2, age^3, birth cohort, sex by birth cohort, birth cohort by age, sex by birth cohort by age, ethnicity, and practice effect. Memory models additionally include birth cohort by age^3 and lower-order interactions. Fluency models additionally include practice effect by sex and birth cohort by sex by age^2 and lower-order interactions. Results are shown for the reference category: participants aged 60 years.
Positive value indicates slower cognitive decline in men.

Table S13. Sex differences in 13-year cognitive decline: Analyses stratified by birth cohort and education, and restricted to follow-up period 2002 to 2015.[a]

ELSA & Whitehall II	Sex difference (95% CI)
**Memory**	
**Education: Below A-level**	
1930-1938	No data
1939-1945	-0·13 (-0·23, -0·03)
1946-1955	-0·14 (-0·24, -0·05)
P sex difference by birth cohort	*0·84*
**Education: A-level and above**	
1930-1938	No data
1939-1945	-0·05 (-0·18, 0·08)
1946-1955	-0·13 (-0·24, -0·02)
P sex difference by birth cohort	*0·37*
**Fluency**	
**Education: Below A-level**	
1930-1938	No data
1939-1945	0·02 (-0·07, 0·11)
1946-1955	0·09 (-0·23, 0·42)
P sex difference by birth cohort	*0·68*
**Education: A-level and above**	
1930-1938	No data
1939-1945	-0·12 (-0·25, 0·00)
1946-1955	0·23 (-0·20, 0·66)
P sex difference by birth cohort	*0·12*

[a]N = 15,368.
Basic models include sex, sex by age, age^2, age^3, birth cohort, sex by birth cohort, birth cohort by age, sex by birth cohort by age, ethnicity, and practice effect. Memory models additionally include birth cohort by age^3 and lower-order interactions. Fluency models additionally include practice effect by sex and birth cohort by sex by age^2 and lower-order interactions. Results are shown for the reference category: participants aged 60 years.
Positive value indicates slower cognitive decline in men.

Table S14. Role of education in sex differences in cognitive performance: Analyses stratified by birth cohort using multiple imputation to account for missing education data.

	At age 50 years		At age 60 years		At age 70 years	
	Basic Model[a]	Basic Model[a] + Education	Basic Model[a]	Basic Model[a] + Education	Basic Model[a]	Basic Model[a] + Education
**ELSA & Whitehall II**	**Sex difference (95% CI)**	**Sex difference (95% CI)**	**Sex difference (95% CI)**	**Sex difference (95% CI)**	**Sex difference (95% CI)**	**Sex difference (95% CI)**
**Memory**						
Birth cohort						
1930-1938	No data	No data	-0·10 (-0·16, -0·03)	-0·24 (-0·31, -0·17)	-0·13 (-0·18, -0·09)	-0·25 (-0·29, -0·20)
1939-1945	No data	No data	-0·12 (-0·16, -0·07)	-0·21 (-0·26, -0·17)	-0·20 (-0·26, -0·15)	-0·29 (-0·34, -0·24)
1946-1955	-0·06 (-0·12, -0·01)	-0·14 (-0·19, -0·08)	-0·17 (-0·22, -0·13)	-0·25 (-0·28, -0·21)	-0·29 (-0·35, -0·23)	-0·35 (-0·41, -0·29)
P sex difference by birth cohort			*0.070*	*0.57*	*0.00040*	*0.014*
**Fluency**						
Birth cohort						
1930-1938	No data	No data	0·19 (0·08, 0·31)	0·04 (-0·08, 0·16)	0·18 (0·13, 0·23)	0·06 (0·01, 0·10)
1939-1945	No data	No data	0·10 (0·04, 0·15)	-0·02 (-0·07, 0·04)	0·03 (-0·02, 0·08)	-0·07 (-0·12, -0·02)
1946-1955	0·06 (-0·01, 0·13)	-0·03 (-0·10, 0·04)	0·00 (-0·05, 0·05)	-0·09 (-0·13, -0·04)	-0·01 (-0·11, 0·08)	-0·08 (-0·17, 0·02)
P sex difference by birth cohort			*0.0018*	*0.034*	*<0.0001*	*0.00040*

[a]Basic models include sex, sex by age, age^2, age^3, birth cohort, sex by birth cohort, birth cohort by age, sex by birth cohort by age, ethnicity, and practice effect. Memory models additionally include birth cohort by age^3 and lower-order interactions. Fluency models additionally include practice effect by sex and birth cohort by sex by age^2 and lower-order interactions.
Positive value indicates male advantage in performance.

Table S15. Role of education in sex differences in 13-year cognitive decline: Analyses stratified by birth cohort using multiple imputation to account for missing education data.

	Basic Model[a]	Basic Model[a] + Education
**ELSA & Whitehall II**	**Sex difference (95% CI)**	**Sex difference (95% CI)**
**Memory**		
Birth cohort		
1930-1938	-0·05 (-0·11, 0·01)	-0·01 (-0·07, 0·06)
1939-1945	-0·12 (-0·18, -0·05)	-0·10 (-0·17, -0·04)
1946-1955	-0·15 (-0·20, -0·09)	-0·14 (-0·20, -0·09)
P sex difference by birth cohort	*0.068*	*0.0074*
**Fluency**		
Birth cohort		
1930-1938	-0·05 (-0·17, 0·08)	-0·01 (-0·13, 0·11)
1939-1945	-0·05 (-0·12, 0·01)	-0·04 (-0·10, 0·03)
1946-1955	0·06 (-0·11, 0·24)	0·09 (-0·08, 0·27)
P sex difference by birth cohort	*0.48*	*0.41*

[a]Basic models include sex, sex by age, age^2, age^3, birth cohort, sex by birth cohort, birth cohort by age, sex by birth cohort by age, ethnicity, and practice effect. Memory models additionally include birth cohort by age^3 and lower-order interactions. Fluency models additionally include practice effect by sex and birth cohort by sex by age^2 and lower-order interactions. Results are shown for the reference category: participants aged 60 years.
Positive value indicates slower cognitive decline in men.
